# Epoetin Theta with a New Dosing Schedule in Anaemic Cancer Patients Receiving Nonplatinum-Based Chemotherapy: A Randomised Controlled Trial

**DOI:** 10.1111/j.1753-5174.2011.00035.x

**Published:** 2011-09

**Authors:** Sergei A Tjulandin, Peter Bias, Reiner Elsässer, Beate Gertz, Erich Kohler, Anton Buchner

**Affiliations:** * Department of Clinical Pharmacology and Chemotherapy, Russian Cancer Research CenterMoscow, Russia; † Department of Clinical ResearchMerckle GmbH, Ulm, Germany; ‡ Department of BiostatisticsMerckle GmbH, Ulm, Germany; § Department of Preclinical and Clinical ResearchBioGeneriX AG, Mannheim, Germany

**Keywords:** Cancer, Epoetin Theta, Placebo, Symptomatic Anaemia

## Abstract

**Introduction:**

Recombinant human erythropoietin (r-HuEPO) is used to treat symptomatic anaemia due to chemotherapy. A new r-HuEPO, Epoetin theta (Eporatio®), was investigated and compared to placebo in a randomised, double-blind clinical trial in adult cancer patients receiving nonplatinum-based chemotherapy. The primary efficacy endpoint was the responder rate (complete haemoglobin (Hb) response, i.e., Hb increase ≥2 g/dl) without the benefit of a transfusion within the previous 4 weeks.

**Research Design and Methods:**

186 patients were randomised to s.c. treatment for 12 weeks with either Epoetin theta (N = 95) or placebo (N = 91). The starting dose was 20,000 IU once weekly Epoetin theta or placebo.

**Results:**

The incidence of complete Hb responders was significantly higher in the Epoetin theta group than in the placebo group (72.6 vs. 25.3%, *P* < 0.0001). More patients in the placebo group than in the Epoetin theta group received blood transfusions after randomisation (23 patients, 25.3% vs. 13 patients, 13.7%, *P* = 0.0277). The majority of patients with a complete Hb response had 20,000 IU/week as their maximum dose prior to response, indicating that a dose of 20,000 IU is an appropriate starting dose. The overall frequencies of adverse events (AEs) were similar in both treatment groups. Hypertension was the only AE that was more frequent in the Epoetin theta group compared to the placebo group (8.4 vs. 1.1%).

**Conclusions:**

Epoetin theta showed a superior efficacy to placebo in terms of complete Hb response without blood transfusion within the previous 4 weeks. Treatment with Epoetin theta resulted in a statistically significant increase in mean haemoglobin levels compared to placebo. The overall frequencies of adverse events were similar in both treatment groups.

## Introduction

Cancer patients may develop anaemia as a result of disease characteristics, chemotherapy or due to decreased endogenous erythropoietin production. Erythropoietin is the essential growth factor required for the production of red blood cells from late progenitor cells of the erythroid lineage in bone marrow. Patients with reduced or absent endogenous erythropoietin production may need to receive exogenous erythropoietin as replacement therapy for the stimulation of erythropoiesis and correction of their symptomatic anaemia.

Anaemia due to chemotherapy is a major clinical problem and a typical later complication of chemotherapy treatment. The aims of management of this condition are to minimise the use of blood or red cell transfusions, to eliminate symptoms arising from anaemia, to improve quality of life (QoL) and to minimise secondary effects of anaemia. Transfusion is the traditional approach to the management of anaemia. However, growing concerns about infection risks, and the fact that the supply of transfusion products is limited has led to a reduction in the use of red cell transfusions, opening up a need for alternatives to transfusion.

Recombinant human erythropoietin has been used in the treatment of anaemia due to chemotherapy for more than 20 years [1,2]. All epoetins are structurally similar and have the same polypeptide receptor binding sites. However, there are subtle differences in the glycosylation patterns of different types of epoetins.

Epoetin theta which was developed by BioGeneriX AG contains recombinant human erythropoietin (r-HuEPO) as the active drug substance and is structurally similar to other recombinant epoetins. The starting dose of Epoetin theta is 20,000 IU subcutaneously (s.c.) once weekly. This starting dose is lower than the recommended starting dose of Epoetin beta (30,000 IU per week) and Epoetin alfa (450 IU/kg per week).

The objective of this study was to demonstrate superiority of Epoetin theta compared to placebo for efficacy during the treatment period of 12 weeks in patients with solid tumours or non-myeloid haematological malignancies receiving nonplatinum chemotherapy.

## Patients and Methods

### Patients

A multinational, multicentre, randomised, placebo-controlled, double-blind phase III study was performed at 72 study centres in 10 countries. The study was conducted according to Good Clinical Practice and the Declaration of Helsinki (1996) and all patients signed informed consent before any study-related activities were performed. The study protocol, amendments, informed consent documents and other relevant study-related documents were reviewed and approved by independent ethics committees of all participating countries (Argentina, Belarus, Brazil, Bulgaria, Chile, India, Moldova, Romania, Russia, Ukraine). Between November 2005 and May 2007 patients with secondary anaemia related to nonplatinum chemotherapy participated in the study. Male and female patients ≥18 years of age with histologically or cytologically proven diagnosis of a solid tumour or non-myeloid haematological tumour were eligible for the study if they gave written informed consent and had anaemia caused by nonplatinum-based chemotherapy defined by a documented Hb concentration of ≤11 g/dl after the last chemotherapy prior to inclusion. Further inclusion criteria among others requested that the patients had at least 1 previous nonplatinum-based chemotherapy cycle as treatment of the current malignancy during the last 4 weeks and had Eastern Cooperative Oncology Group (ECOG) performance status 0, 1, 2 or 3. Exclusion criteria were any other primary haematologic disorder that would cause anaemia, head and neck tumours, uncontrolled severe hypertension and concomitant radiotherapy. Iron substitution was allowed during the study.

### Study Design

**Figure 1:**
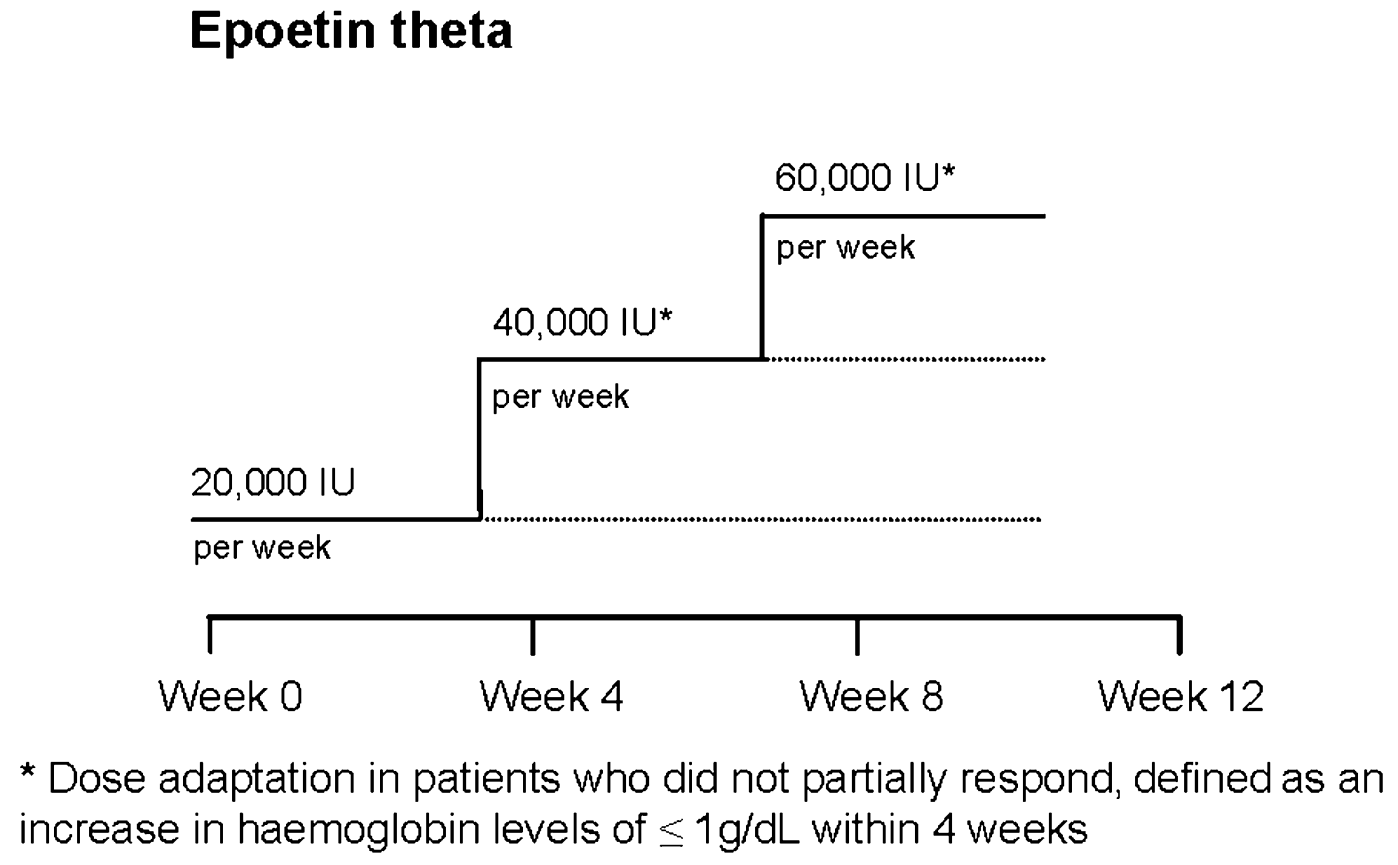
Dose adaptation of Epoetin theta.

A total of 186 patients were randomised using a computer-generated allocation schedule in a 1:1 ratio stratified by country to double blind treatment for 12 weeks with either Epoetin theta (N = 95) or placebo (N = 91). All persons involved in the conduct of the study were blinded with respect to the study medication. Recruitment period was 15 months and the study ended regularly. Patients randomised to Epoetin theta received a starting dose of 20,000 IU Epoetin theta subcutaneously (s.c.) once weekly or the same volume of placebo. This starting dose was increased to 40,000 IU/week in patients who did not have a partial response (Hb increase of ≥1 g/dl) after 4 weeks of treatment, and again to 60,000 IU/week in case of insufficient response after the second 4 week period of treatment (Figure 1). If the patient's Hb level increased by more than 2 g/dl in a 4-week period the weekly dose was reduced by 50%. If the Hb level exceeded 13 g/dl the dose was reduced to 50% of the recent dose or was temporarily withhold. The selection of the Epoetin theta doses was based on recent evidence that lower doses than those recommended for authorised products may achieve a comparable Hb response, that the same weekly doses administered once or three times weekly result in the same Hb response [3–5] and that fixed doses rather than weight-based doses can be used in anaemic cancer patients [6]. Patients randomised to placebo received s.c. injections of placebo and dose adjustment according to the same schedule as for Epoetin theta for blinding purposes.

Hb, haematocrit and reticulocytes were measured weekly throughout the study. Baseline levels were defined as the mean of two values measured prior to initiation of drug treatment. The FACT-An questionnaire including FACT-G and -F [7] was used to assess the QoL.

Immunogenicity was assessed by a predefined cascade of antibody assays. This cascade was structured into a sequential scheme comprising screening, confirmation and characterisation of clinical specimens. Confirmed positive samples were investigated for neutralising antibodies in a cellular assay using an erythropoietin dependent UT-7 cell line.

Epoetin theta and placebo were provided in glass vials. The study drug was administered by appropriately qualified study personnel. The investigator and all other study personnel were kept blinded and performed all assessments of the patient without knowledge of treatment. An unblinded independent Data Safety Monitoring Committee closely monitored the safety in order to ensure that patients were not exposed to an unjustifiable risk.

### Endpoints

The pre-specified primary endpoint was the number of patients with a complete Hb response which was defined as an increase in Hb of ≥2 g/dl from baseline without the benefit of a transfusion within the previous four weeks. Secondary efficacy endpoints included the number of patients having a partial Hb response (defined as increase of ≥1 g/dl from baseline), the number of patients having a complete Hb response with the starting dose, the number of patients having a partial Hb response with the starting dose, the number of patients receiving transfusions, the number of blood units transfused, the time course of Hb, haematocrit and reticulocytes, the QoL (FACT-An, including FACT-G and -F [7]), and the dose of Epoetin theta at the time of complete/partial Hb response. Blood transfusions were administered on a case by case basis at the discretion of the investigator. Transfusions should have been avoided at a haemoglobin level ≥8.5 g/dl. Safety endpoints included safety lab, vital signs, incidence of adverse events (AEs) and adverse drug reactions (ADRs), incidence of hypertension, overall tolerability and screening for anti-drug antibodies to Epoetin theta at the beginning and end of the study.

### Statistical Methods

A logistic regression analysis with treatment and type of cancer as explanatory and baseline haemoglobin value as continuos variables was performed to estimate the difference in the proportion of complete haemoglobin responders between Epoetin theta and placebo in the confirmatory analysis of the primary efficacy endpoint. For the other binary secondary efficacy endpoints the same logistic regression model as for the primary endpoint was estimated. Changes of QoL (FACT-scores) from baseline to end of study were compared pairwise with the Wilcoxon-Mann-Whitney test, whereas the treatment groups for the other secondary efficacy endpoints were only compared descriptively. Descriptive *P*-values were calculated with appropriate statistical tests but were regarded as supportive only. For the primary efficacy endpoint a subgroup analysis with type of malignancy (solid, non-myeloid haematological) was performed. All descriptive tables were displayed by country.

The sample size calculation required the inclusion of 80 patients per treatment group to achieve a power of 90% for the statistical superiority test comparing Epoetin theta and placebo assuming a response rate of 45% for Epoetin theta and 20% for placebo.

## Results

**Figure 2:**
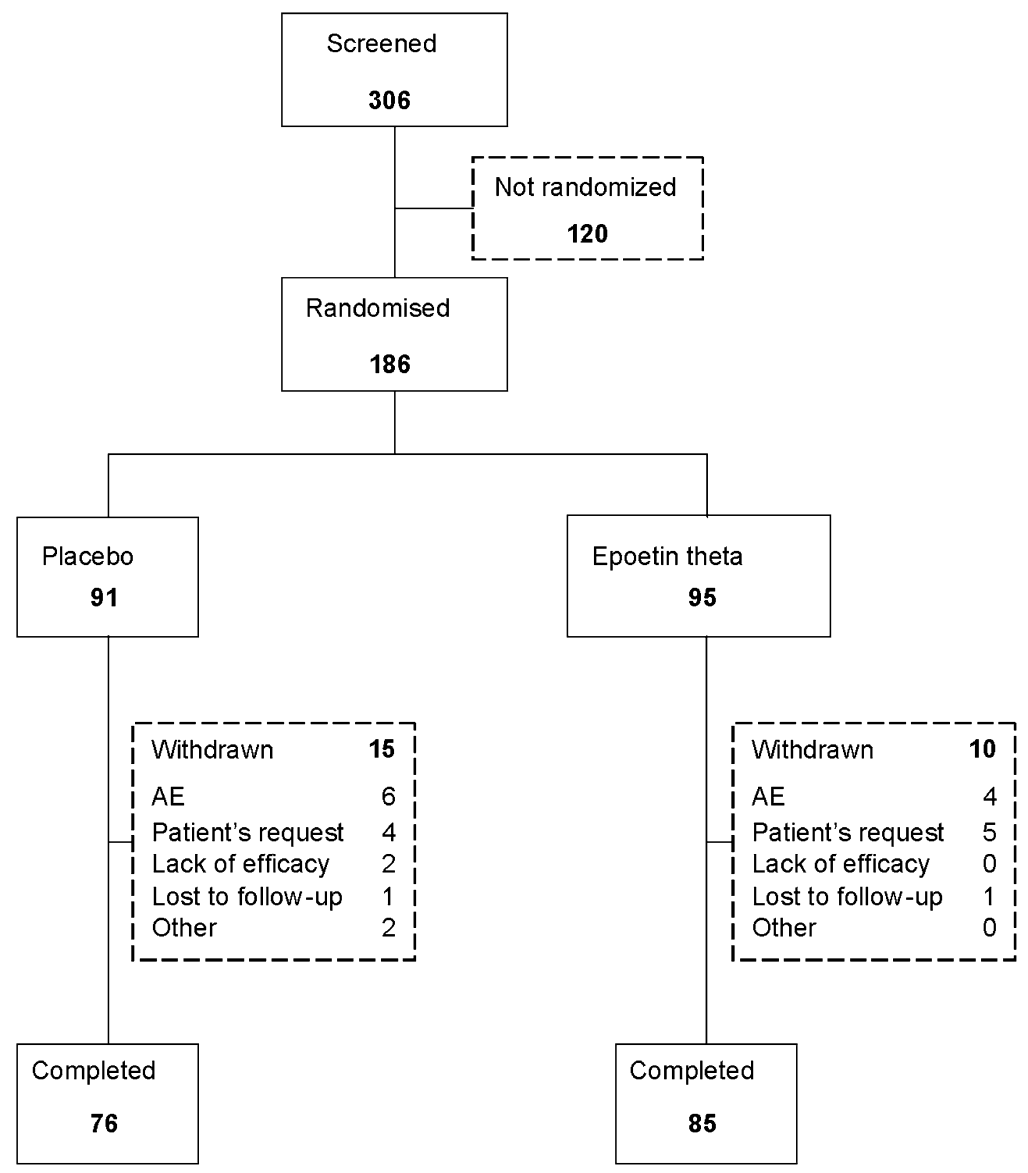
Disposition of patients.

The demographic and baseline characteristics were comparable across the 2 treatment groups. There were no relevant differences between treatment groups with regard to medical history, prior or concomitant medications, ECOG performance status, previous chemotherapy, concomitant diseases, and primary malignant disease (Table 1). There were no clinically noteworthy differences between the treatment groups with regard to on-study chemotherapies. In total, 161 (86.6%) patients completed the study according to protocol (Figure 2). Of the 25 patients who prematurely discontinued the study, 15 were in the placebo group and 10 in the Epoetin theta group. The most common reason for discontinuation was the occurrence of an AE (10 patients), followed by patient's request (9 patients).

**Table 1:** Patient characteristics

	Placebo (n = 91)	Epoetin theta (n = 95)	Total (n = 186)
Gender [n (%)]			
Male	34 (37.4%)	30 (31.6%)	64 (34.4%)
Female	57 (62.6%)	65 (68.4%)	122 (65.6%)
Age [years]			
Mean ± SD	55.8 ± 14.3	56.9 ± 14.7	56.3 ± 14.5
Median	57.0	60.0	58.0
Range	18.0 to 82.0	18.0 to 83.0	18.0 to 83.0
Body weight [kg]			
Mean ± SD	68.6 ± 14.1	67.4 ± 15.2	68.0 ± 14.6
Median	68.0	68.0	68.0
Range	41.0 to 103.0	43.0 to 127.0	41.0 to 127.0
ECOG performance status			
0	9 (9.9%)	14 (14.7%)	23 (12.4%)
1	60 (65.9%)	53 (55.8%)	113 (60.8%)
2	21 (23.1%)	28 (29.5%)	49 (26.3%)
3	1 (1.1%)	0	1 (0.5%)
Most common malignancies			
Multiple myeloma	17 (18.7%)	19 (20.0%)	36 (19.4%)
Breast cancer	17 (18.7%)	16 (16.8%)	33 (17.7%)
Chronic lymphocytic leukaemia	7 (7.7%)	5 (5.3%)	12 (6.5%)
Gastric cancer	3 (3.3%)	6 (6.3%)	9 (4.8%)
Most common on-study CT			
Cyclophosphamide	47 (51.6%)	50 (52.6%)	97 (52.2%)
Doxorubicin	29 (31.9%)	32 (33.7%)	61 (32.8%)
Vincristine	28 (30.8%)	26 (27.4%)	54 (29.0%)
Dexamethasone	21 (23.1%)	22 (23.2%)	43 (23.1%)
Prednisolone	26 (28.6%)	14 (14.7%)	40 (21.5%)

Abbreviations: n = number of patients; SD = standard deviation; CT = chemotherapy.

**Figure 3:**
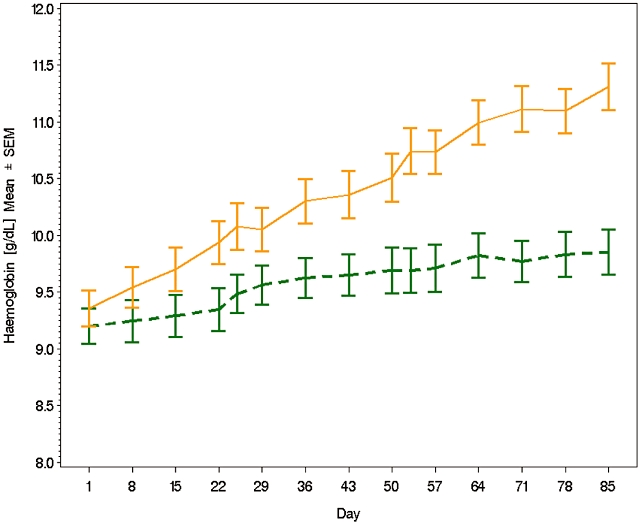
Time course of mean (±SEM) haemoglobin values for the full analysis set. Epoetin theta (yellow) and placebo (green).

The results of efficacy endpoints for the full analysis set are summarised in Table 2 (number of patients with a complete and partial Hb response without blood transfusion, number of patients with a complete and partial Hb response without blood transfusion and with the starting dose and number of patients receiving blood transfusions) and Figure 3 (time course of Hb).

**Table 2:** Results of efficacy endpoints. Number of patients with a complete and partial Hb response without blood transfusion, number of patients with a complete and partial Hb response without blood transfusion and with the starting dose and number of patients receiving blood transfusions for the full analysis set

	Placebo (n = 91)	Epoetin theta (n = 95)
Baseline Hb [g/dl] mean ± standard deviation	9.1 ± 1.3	9.2 ± 1.3
Complete Hb response without blood transfusion:		
N (%)	23 (25.3%)	69 (72.6%)
*P* value	<0.0001	
Odds ratio (95% CI)	7.944 (4.182, 15.632)	
Complete Hb response without blood transfusion and dose adjustment:		
N (%)	9 (9.9%)	43 (45.3%)
*P* value	<0.0001	
Odds ratio (95% CI)	7.728 (3.590, 18.285)	
Partial Hb response without blood transfusion:		
N (%)	56 (61.5%)	78 (82.1%)
*P* value	0.0025	
Odds ratio (95% CI)	2.841 (1.462, 5.694)	
Partial Hb response without blood transfusion and dose adjustment:		
N (%)	24 (26.4%)	56 (58.9%)
*P* value	<0.0001	
Odds ratio (95% CI)	4.028 (2.179, 7.632)	
Patients received blood transfusions		
N (%)	23 (25.3%)	13 (13.7%)
*P* value	0.0277	
Odds ratio (95% CI)	0.352 (0.133, 0.868)	

Abbreviations: n = number of patients; Hb = haemoglobin; CI = confidence interval.

### Extent of Exposure

The mean ± SD average weekly dose was 25,905 ± 10,956 IU in the Epoetin theta group. The mean treatment duration ± SD was comparable in both groups (71.9 ± 16.9 days placebo vs. 72.1 ± 15.7 days Epoetin theta).

### Complete Haemoglobin Response Without Blood Transfusion

The primary parameter for the confirmatory analysis was the complete Hb response (increase of ≥2 g/dl from baseline) without blood transfusion. The responder rate in the Epoetin theta group was substantially higher than in the placebo group (72.6 vs. 25.3%). The difference between Epoetin theta and placebo was statistically significant (*P* < 0.0001) with a baseline Hb adjusted odds ratio (OR) of 7.94 (95% CI: 4.18, 15.63) (Table 2). Type of cancer and baseline haemoglobin levels had no statistically significant effects on the response rate.

The proportion of patients who had a complete Hb response without changing the starting dose was significantly higher (*P* < 0.0001, OR: 7.73, 95%CI: 3.59, 18.29) in the Epoetin theta group than in the placebo group (45.3 vs. 9.9%) (Table 2). Type of cancer and baseline haemoglobin levels had also no statistically significant effects on the response rate.

### Partial Haemoglobin Response Without Blood Transfusion

The partial Hb response was defined as increase of ≥1 g/dl from baseline without blood transfusion within the last four weeks. As for the complete Hb response, the responder rate was higher with Epoetin theta than with placebo (82.1 vs. 61.5%, *P* = 0.0025, OR: 2.84, 95% CI: 1.46, 5.69) (Table 2). Type of cancer and baseline haemoglobin levels had no statistically significant effects on the response rate. The proportion of patients who had a partial Hb response without blood transfusion and without changing the starting dose was significantly higher (*P* < 0.0001, OR: 4.03, 95% CI: 2.18, 7.63) in the Epoetin theta group compared to placebo (58.9 vs. 26.4%) (Table 2). Type of cancer and baseline haemoglobin levels had also no statistically significant effects on the partial response rate.

### Number of Patients Receiving Blood Transfusions

More patients in the placebo group than in the Epoetin theta group received blood transfusions after randomisation (25.3 vs. 13.7%). The difference between the treatment groups was statistically significant (*P* = 0.0277) with an odds ratio of: 0.35 (95% CI: 0.13, 0.87) (Table 2). Baseline haemoglobin levels had a statistically significant effect on the rate of blood transfusion (*P* < 0.0001) with an odds ratio of 0.32 (95% CI: 0.21, 0.47) per g/dl baseline Hb. Type of cancer had no statistically significant effect on the rate of blood transfusion.

### Number of Blood Units Transfused

The mean (±SD) number of blood units transfused after randomisation in those patients who needed a transfusion was 4.1 ± 2.8 units in the placebo group and 3.5 ± 3.5 units in the Epoetin theta group.

### Time Course of Haemoglobin, Haematocrit and Reticulocytes

Baseline Hb values were similar in both treatment groups (Figure 3). Over the course of the study mean Hb levels rose steadily in the Epoetin theta group, exceeding 10 g/dl by day 25 and reached 11 g/dl by day 64. The mean ± SD Hb levels rose from 9.2 ± 1.3 g/dl at baseline to 11.3 ± 2.0 g/dl at the end of the study, resulting in an increase of 2.1 g/dl. In the placebo group, a slight rise in mean haemoglobin levels was observed over the course of the study but the mean level never reached 10 g/dl. Treatment with Epoetin theta resulted in a statistically significant (*P* < 0.0001) increase in mean haemoglobin levels by the end of the study compared to placebo.

The changes of haematocrit values were very similar to the changes of Hb values over time. Absolute reticulocyte values showed a high degree of variability in both treatment groups and at all timepoints.

### Dose of Epoetin Theta at the Time of Partial or Complete Haemoglobin Response

The mean ± SD weekly dose of Epoetin theta at the time of complete Hb response without blood transfusion was 27,681.2 ± 14,260.7 IU (median 20,000 IU), and at the time of partial Hb response it was 24,871.8 ± 10,659.3 IU (median 20,000 IU). The mean dose of Epoetin theta at the time of complete and partial Hb response was similar for solid tumours and haematological malignancies. A dose of up to 20,000 IU/week was sufficient for complete Hb response in 66.7% of patients with complete response in the Epoetin theta group. In a further 23.2% of patients with complete response, response was achieved with a dose of 40,000 IU/week.

### Quality of Life

The completion rate of valid FACT-An questionnaires was high in both treatment groups: 89.5 to 97.9% in the Epoetin theta and 85.7 to 96.7% in the placebo group with only small decreases in completion rates observed over the course of the study in both groups. For all scores mean changes from baseline were in favour of the Epoetin theta group compared to placebo, however the differences were not statistically significant, considering that the study was not powered for this comparison (Table 3).

**Table 3:** Mean changes from baseline in FACT scores

Score type	Placebo (n = 91) N valid = 84	Epoetin theta (n = 95) N valid = 88
FACT-An Total		
Mean ± SD	0.6 ± 22.0	6.3 ± 21.7
*P* value	*P* = 0.243	
FACT-An Trial outcome index		
Mean ± SD	1.2 ± 18.8	5.6 ± 17.1
*P* value	*P* = 0.222	
FACT-F Fatigue module		
Mean ± SD	0.6 ± 8.8	2.9 ± 7.9
*P* value	*P* = 0.142	
FACT-G Total score		
Mean ± SD	−0.2 ± 12.4	3.0 ± 12.7
*P* value	*P* = 0.224	

Abbreviations: n = number of patients; N valid = number of valid questionnaires; SD = standard deviation; FACT-An = Functional Assessment of Cancer Therapy—Anaemia scale; FACT-F = Functional Assessment of Cancer Therapy—Fatigue scale; FACT-G = Functional Assessment of Cancer Therapy—General scale.

### Safety Evaluation

The overall frequencies of adverse events (AEs) were similar in both treatment groups with 78.0% in the placebo group and 80.0% in the Epoetin theta group (Table 4). The high incidence of AEs was to be expected in cancer patients receiving chemotherapy. The overall profile of the most frequently reported AEs can be expected in the rather elderly study population of cancer patients receiving nonplatinum chemotherapy. Frequencies only exceeded 10% for asthenia (20.4%), neutropenia (18.8%), nausea (17.2%), leukopenia (15.6%) and pyrexia (12.9%). Hypertension was the only AE that was more frequent in the Epoetin theta group compared to the placebo group (8.4 vs. 1.1%). Hypertension is the most frequently reported side effect of treatment with epoetins. Skin reactions that might have been caused by the subcutaneous administration of study medication were reported in 20 patients (13 Epoetin theta, 13.7% and 7 placebo, 7.7%). None of the skin reactions was severe or serious.

**Table 4:** Frequencies of AE categories (full analysis set)

	Placebo (n = 91)		Epoetin theta (n = 95)		Total (n = 186)	
			
Category of AE	n	%	n	%	n	%
Any AE	71	78.0	76	80.0	147	79.0
Related AE = ADR	18	19.8	27	28.4	45	24.2
Serious AE	14	15.4	11	11.6	25	13.4
Serious ADR	1	1.1	0	—	1	0.5
Death	5	5.5	6	6.3	11	5.9

Abbreviations: n = number of patients; multiple mentions per patient are possible.

Adverse drug reactions (ADRs) with a causal relationship to the study medication as assessed by the investigator were reported in 27 (28.4%) patients in the Epoetin theta group and 18 (19.8%) patients in the placebo group (Table 4). The most common ADRs were asthenia (7.5%), nausea (5.4%), headache (3.2%), pyrexia (2.7%) and vomiting (2.2%). All of these events commonly occur in cancer patients receiving chemotherapy.

Eleven of the patients in this study (6 Epoetin theta, 5 placebo) died during the study period (Table 4). The most frequent reason for death was disease progression (3 placebo, 2 Epoetin theta). Serious adverse events (SAEs) were reported in 25 (13.4%) patients (11 Epoetin theta, 14 placebo) (Table 4). Just 1 of the SAEs (pyrexia in a placebo patient) was assessed as related to the study medication. AEs leading to study discontinuation were reported in 10 patients (4 Epoetin theta, 6 placebo) and 1 patient in the placebo group discontinued due to a ADR (thrombophlebitis).

Results for safety lab variables, vital signs, body weight, 12-lead ECG, physical examination, tolerability, skin irritation, and results of current chemotherapy did not give rise to any safety concerns.

Tolerability as assessed by the patients was very good or good in 89.5% and 89.0% of patients in the Epoetin theta and placebo group, respectively. The investigators assessed tolerability as very good or good in 98.9% (Epoetin theta) and 96.7% (placebo) of the patients.

The response rate (complete plus partial response) to chemotherapy was slightly higher in the Epoetin theta group (45.3 vs. 40.7%), therefore there is no evidence that Epoetin theta could have had a negative impact on the course of the disease or on the efficacy of the chemotherapy.

The incidence of anti-drug antibodies to Epoetin theta was assessed at the beginning and end of the study. Only 1 patient treated with placebo developed a single positive result at baseline. A cellular assay to detect neutralisation was negative and a blood sample taken from this placebo-treated patient at the end of the study was also negative. None of the patients enclosed in the study developed neutralising anti-epoetin antibodies to Epoetin theta.

## Discussion

The selection of the weekly fixed starting dose of 20,000 IU Epoetin theta, which is lower than the recommended starting dose of other epoetins, is in line with recent recommendations for the treatment of cancer patients with epoetins, i.e., to use the lowest dose needed to avoid red blood cell transfusion [8].

A meta-analysis based on 14 randomised controlled trials including 2,347 cancer patients, with haematologic response to treatment with erythropoietin defined as an increase in Hb of at least 2 g/dl unrelated to transfusion, reported response rates of 9–70% [9]. The corresponding relative risk (RR) for haematologic response in the erythropoietin group was 3.60 (95% CI: 3.07, 4.23). Thus, patients receiving an erythropoietin are 3 to 4 times more likely to achieve Hb response. The response rates of 72.6% in the Epoetin theta group and 25.3% in the placebo group observed in this study therefore is in line with the literature, although the weekly starting dose was fixed and not body weight adjusted and lower than the weekly dose in the studies included in the above meta-analysis.

A key clinical factor in the treatment of anaemia due to chemotherapy is reduction in the need for blood transfusions. A meta-analysis based on 25 randomised controlled trials including 3,069 cancer patients showed that the use of erythropoietin significantly reduced the relative risk of red blood cell transfusion (RR 0.67, 95% CI: 0.62, 0.73) [9]. In the present study, a significantly lower proportion of patients in the Epoetin theta group than in the placebo group received blood transfusions after randomisation (13.7 vs. 25.3%, *P* = 0.0277).

The results of the present study is in line with information derived from the literature. This holds true also for the further efficacy parameters like the haemoglobin and haematocrit levels over time. A total of 66.7% of the complete responders in the Epoetin theta group achieved the response with the starting dose of 20,000 IU/week. This confirms that the Epoetin theta dosing schedule tested here is suitable for an effective and well tolerated treatment of anaemic cancer patients.

The frequencies and types of AEs as observed in this study were to be expected in cancer patients receiving chemotherapy. Furthermore, no anti-drug antibodies to Epoetin theta were detected in any patient of this study.

Results for other safety parameters like SAEs, AEs leading to discontinuation, safety lab variables, vital signs, etc. did not give rise to any safety concerns.

## Conclusions

Epoetin theta with a weekly starting dose of 20,000 IU is superior to placebo in terms of complete Hb response without blood transfusion. In this small 12 week study of 186 patients, it was shown that Epoetin theta is a safe and effective treatment for anaemia due to nonplatinum chemotherapy in patients with solid tumours or non-myeloid haematological malignancies.

## References

[1] Eschbach JW, Egrie JC, Downing MR, Browne JK, Adamson JW (1987). Correction of the anemia of end-stage renal disease with recombinant human erythropoietin: Results of a combined phase I and II clinical trial. N Engl J Med.

[2] Winearls CG, Oliver DO, Pippard MJ, Reid C, Downing MR, Cotes PM (1986). Effect of human erythropoietin derived from recombinant DNA on the anaemia of patients maintained by chronic haemodialysis. Lancet.

[3] Cazzola M, Messinger D, Battistel V, Bron D, Cimino R, Enller-Ziegler L (1995). Recombinant human erythropoietin in the anemia associated with multiple myeloma or non-Hodgkin's lymphoma: Dose finding and identification of predictors of response. Blood.

[4] Cazzola M, Beguin Y, Kloczko J, Spicka I, Coiffier B (2003). Once-weekly epoetin beta is highly effective in treating anaemic patients with lymphoproliferative malignancy and defective endogenous erythropoietin production. Br J Haematol.

[5] Granetto C, Ricci S, Martoni A, Pezzella G, Testore F, Mattioli R (2003). Comparing the efficacy and safety of fixed versus weight-based dosing of epoetin alpha in anemic cancer patients receiving platinum-based chemotherapy. Oncol Rep.

[6] Bokemeyer C, Aapro MS, Courdi A, Foubert J, Link H, Osterborg A (2004). EORTC guidelines for the use of erythropoietic proteins in anaemic patients with cancer. Eur J Cancer.

[7] Cella D (1997). The functional assessment of cancer therapy-anaemia (FACT-AN) scale: A new tool for the assessment of outcomes in cancer anaemia and fatigue. Semin Hematol.

[8] European Medicines Agency (2007). EMEA Public statement: European medicines agency starts review of the safety of epoetins.

[9] Bohlius J, Weingart O, Trelle S, Engert A (2006). Cancer-related anemia and recombinant human erythropoietin—An updated overview. Nature Clin Pract Oncol.

